# Accurate Determination of the Oxidative Phosphorylation Affinity for ADP in Isolated Mitochondria

**DOI:** 10.1371/journal.pone.0020709

**Published:** 2011-06-09

**Authors:** Gilles Gouspillou, Richard Rouland, Guillaume Calmettes, Véronique Deschodt-Arsac, Jean-Michel Franconi, Isabelle Bourdel-Marchasson, Philippe Diolez

**Affiliations:** 1 Laboratoire de Résonance Magnétique des Systèmes Biologiques, UMR 5536 CNRS - Université Victor Segalen Bordeaux 2, Bordeaux, France; 2 CHU de Bordeaux - Pôle de gérontologie clinique, Hôpital Xavier Arnozan, Pessac, France; Instituto de Química - Universidade de São Paulo, Brazil

## Abstract

**Background:**

Mitochondrial dysfunctions appear strongly implicated in a wide range of pathologies. Therefore, there is a growing need in the determination of the normal and pathological integrated response of oxidative phosphorylation to cellular ATP demand. The present study intends to address this issue by providing a method to investigate mitochondrial oxidative phosphorylation affinity for ADP in isolated mitochondria.

**Methodology/Principal Findings:**

The proposed method is based on the simultaneous monitoring of substrate oxidation (determined polarographically) and phosphorylation (determined using the glucose - hexokinase - glucose-6-phosphate dehydrogenase - NADP^+^ enzymatic system) rates, coupled to the determination of actual ADP and ATP concentrations by bioluminescent assay. This enzymatic system allows the study of oxidative phosphorylation during true steady states in a wide range of ADP concentrations. We demonstrate how the application of this method allows an accurate determination of mitochondrial affinity for ADP from both oxidation (K_mVox_) and phosphorylation (K_mVp_) rates. We also demonstrate that determination of K_mVox_ leads to an important overestimation of the mitochondrial affinity for ADP, indicating that mitochondrial affinity for ADP should be determined using phosphorylation rate. Finally, we show how this method allows the direct and precise determination of the mitochondrial coupling efficiency. Data obtained from rat skeletal muscle and liver mitochondria illustrate the discriminating capabilities of this method.

**Conclusions/Significance:**

Because the proposed method allows the accurate determination of mitochondrial oxidative phosphorylation affinity for ADP in isolated mitochondria, it also opens the route to a better understanding of functional consequences of mitochondrial adaptations/dysfunctions arising in various physiological/pathophysiological conditions.

## Introduction

In most tissues, such as skeletal muscle, mitochondria are the main site of energy production through the oxidative phosphorylation pathway. In addition to their crucial role in energy metabolism, mitochondria also take part into other important physiological functions such as calcium homeostasis, apoptosis signaling and ROS production. All these roles are in complex and close interaction [1], making mitochondria a key organelle for cell life and death. It is therefore not surprising that the scientific interest surrounding mitochondria is currently growing. Dysfunctions of mitochondrial oxidative phosphorylation are increasingly investigated to define and understand the very mechanisms involved in a large number of pathologies. We [2] and others [3], [4], [5] have shown that mitochondrial dysfunctions are implicated in the aging process and in age-related pathologies such as Alzeimer's [6], Parkinson's and Hungtington's diseases and Friedreich's ataxia [7].

 However, the identification of the functional consequences of mitochondrial dysfunctions on cellular energetics remains a scientific challenge. Indeed, most studies focus on isolated parts of mitochondrial oxidative phosphorylation, making the link between mitochondrial dysfunctions and their consequences on cellular energetics difficult to decipher. It is also well established in the field of systems biology that the emergence of unexpected properties can arise from the cooperative interactions between individual components of a system [8]. Consequently, the understanding of the functional consequences of mitochondrial oxidative phosphorylation dysfunctions on cellular energetics could also benefit from considering the integrated response of oxidative phosphorylation to cellular ATP demand. Among useful parameters of mitochondrial bioenergetics, the mitochondrial coupling efficiency and the mitochondrial affinity for ADP are in this context of particular interest. Mitochondrial affinity for ADP is closely related to the mitochondrial capacity to respond to changes in cellular ATP demand. This parameter is usually determined in the literature by the calculation of apparent Km of respiration for ADP using either isolated mitochondria [9], [10], [11], [12], [13] or permeabilized cells [14], [15]. Indeed, for technical reasons, apparent Km for ADP is most of the time determined from the kinetic response of the respiratory chain activity (oxygen consumption) to the increase in ADP concentration. However, this affinity primarily relies on the response of the ATP production system to this increase in ADP concentration. As respiratory chain activity is not directly linked to phosphorylation activity due to the existence of proton leaks [16], [17], well known to vary depending on mitochondrial activity, the apparent Km for ADP is approximated using these methods and should therefore be determined using phosphorylation rate. As compared to isolated mitochondria, the use of permeabilized cells more closely mimic the physiological conditions, since it allows the study oxidative phosphorylation without disrupting physical interactions of mitochondria with their surrounding environment [18]. However, in permeabilized cells, the phosphorylation rate can only be indirectly determined under highly specific conditions using this approach [19], preventing the accurate determination of mitochondrial phosphorylation affinity for ADP.

 The aim of the present paper is to propose a new and easy to use method allowing a true and accurate determination of oxidative phosphorylation affinity for ADP using isolated mitochondria. This method is based on the direct and simultaneous independent monitoring of oxidation and phosphorylation rates during true steady states under various ADP concentrations (from very low (7 µM) to saturating ADP concentrations), the actual ADP and ATP concentrations being determined by bioluminescent assays. We demonstrate how this method can provide other useful data about the energetics of mitochondria in the cell, since its application also allows the direct determination of mitochondrial coupling efficiency. To achieve this aim, mitochondria isolated from muscle and liver, two populations known to present significant differences, were studied in order to validate the method.

## Materials and Methods

### 1. Ethics Statement

All experiments were conducted in agreement with the National and European Research Council Guide for the care and use of laboratory animals in accordance with the recommendations of the Weatherall report, “The use of non-human primates in research”. All animal protocols used were approved by our local ethics committee named Direction départementale des services vétérinaires de la Gironde, the corresponding license number being P.D. 3308010 (11/19/2008).

### 2. Isolation of liver and skeletal muscle mitochondria

Isolation of liver and skeletal muscle mitochondria from 6 month-old male Wistar rats were anesthetized by isofluran inhalation and killed by intraperitoneal injection of pentobarbital (60 mg.kg^−1^). The gastrocnemius muscle and the liver were then dissected and washed separately in the isolation medium containing 100 mM saccharose, 180 mM KCl, 50 mM Tris, 5 mM MgCl2, 10 mM EDTA and 0.1% (w/v) BSA (pH 7.2). Before homogenization, gastrocnemius muscle was minced and exposed during 5 minutes to protease (2 mg of bacterial proteinase type XXIV per ml of isolation medium, Sigma-Aldrich: P8038). Mitochondria were extracted as described in [2], [20].

### 3. Protein content determination

Mitochondrial protein concentration was determined by the Bradford method [21] using BSA as standard.

### 4. Simultaneous monitoring of oxidation and phosphorylation rates during steady states

Oxygen consumption and ATP synthesis rate were monitored simultaneously in a glass vessel (final volume 6 ml, 25°C) in a medium containing 240 mM Manitol, 100 mM KCl, 1 mM EGTA, 20 mM MgCl_2_, 10 mM KH_2_PO_4_ and 0.1% (w/v) BSA (pH 7.2). Glutamate (5 mM)+Malate (1 mM)+succinate (5 mM) were used as substrates in all experiments.

Oxidation rates were determined polarographically with a Clark electrode (Rank Brothers). O_2_ concentration in air-equilibrated medium was taken as 240 µM [22]. The electrode was connected to a PowerLab (ADInstrument) for data collection.

**Figure 1:**
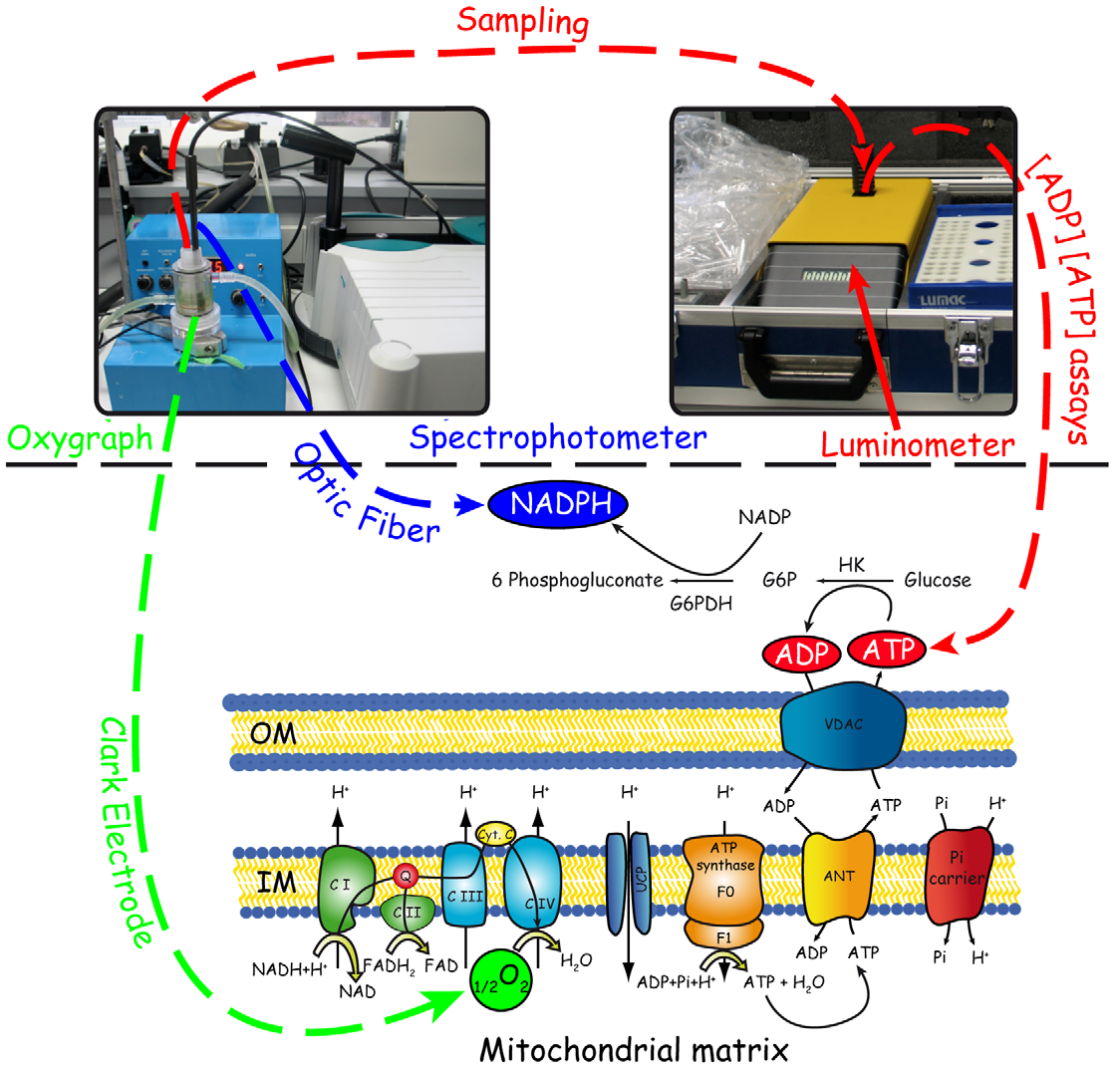
Experimental determination of oxidation rate, phosphorylation rate and ADP/ATP concentrations. Our experimental set-up was composed of an oxygraph, a spectrophotometer and a luminometer. An optic fiber, connected to the spectrophotometer, was inserted in the oxygraphic vessel (picture on the top-left hand corner). Mitochondrial oxidation rate was determined using the Clark electrode of the oxygraph. Phosphorylation rate was assessed, with the help of the optic fiber, by the continuous monitoring of NADPH production in the oxygraphic vessel. Samplings were performed at the onset and the end of the recording to assess both ADP and ATP concentrations using a bioluminescence-based assay with the help of a luminometer (picture on the top-right hand corner). For clarity, all parameters that were measured during each experiment are highlighted by colored circles. HK: hexokinase, G6PDH: glucose 6 phosphate dehydrogenase, G6P: glucose 6 phosphate, OM: outer membrane, IM: inner membrane.

Phosphorylation rates were determined using a coupled enzymatic system composed of Glucose (5 mM) - Hexokinase (2.5 U.ml^−1^, Sigma-Aldrich, H4502) - Glucose-6-phosphotate dehydrogenase (2.5 U.ml^−1^, Sigma-Aldrich, G6378) - NADP^+^ (1.6 mM) [23] (figure 1). Chemicals composing this enzymatic system were in excess in order to avoid any limitation of mitochondrial activity. By this system, the ADP phosphorylated by mitochondria into ATP is regenerated by the phosphorylation of glucose into glucose-6-phosphate (G6P) catalyzed by Hexokinase (HK) (eq. 1). This first reaction allows the establishment of a constant ATP turnover ensuring the study of oxidative phosphorylation during steady states. Resulting G6P is then oxidized by Glucose-6-phosphotate dehydrogenase (G6PDH) to form 6-phosphogluconate, using NADP^+^ as electron acceptor (eq. 2). ATP
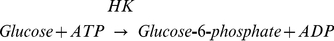
(1)

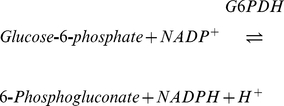
(2)


Consequently, NADPH production is stoichiometrically linked to mitochondrial ATP synthesis rate, which can therefore be monitored by spectrometrically measuring the increase in the absorbance of NADPH at 340 nm. Conversion of NADPH absorbance into NADPH concentration was performed using a molar extinction coefficient of 6.22 mM^−1^.cm^−1^. Changes in rate of NADPH production were therefore monitored with an optic fiber connected to a spectrometer placed in the oxygraphic vessel (Cary 50, Varian). Mitochondrial protein concentration in the oxygraphic vessel for each recording was 25 µg.ml^−1^ in order to decrease mitochondrial non-specific absorption at 340 nm.

 To avoid any interference by residual adenylate kinase activity, an excess of P1,P5- Di(adenosine-5)pentaphosphate (AP5A, 20 µM) was added to the measurement medium in all experiments.

### 5. Determination of ADP and ATP concentrations

ADP and ATP concentrations were determined both at the onset of steady state and just before the end of each recording to assess the stability of nucleotides concentrations. The following protocol was optimized for concentrations ranging from 5 to 50 µM corresponding to the sensitivity range of the ATP monitoring reagent used (FLAA-1KT, Sigma-Aldrich) diluted 625 times:

#### Step 1. Baseline assay

150 µl of the ATP monitoring reagent were added to a luminometer cuvette. Cuvette was then placed in the luminometer chamber (Lumac Biocounter M1500, Lumac) to record the emitted light by the reagent alone. This value was subtracted to the value obtained during the step 2 for accurate determination of ATP concentration.

#### Step 2. Determination of ATP concentration

10 µl of the measured medium were taken in the oxygraphic vessel using a 10 µl Hamilton syringe and instantaneously added to the cuvette prepared during step 1. The amount of emitted light gave ATP concentration in the sample.

#### Step 3. Determination of ADP concentration

Phosphoenol pyruvate (6.5 mM final) and pyruvate kinase (54 U.ml^−1^ final) were then added to the luminometer cuvette in order to convert all ADP into ATP. Subtracting the ATP concentration determined during step 2 allowed the determination of the ADP concentration in the assay.

#### Step 4. Calibration of luminometer response

2.4 µM of a standard solution of ATP was systematically added in the cuvette in order to converter the emitted light by the luciferin-luciferase reaction into µM of ATP.

After each assay, phosphoenol pyruvate and pyruvate kinase were added to the ATP monitoring reagent alone to determine the baseline of light emission under these conditions. This separate assay was used to correct emitted light obtained during steps 3 and 4.

For experiments where the added ADP concentration at the onset of the recording was higher than 50 µM, the 10 µl of the measurement medium taken in the oxygraphic vessel were diluted in water in order to bring the nucleotides concentration between 10 and 50 µM. 10 µl of this diluted solution were then used to determine ADP and ATP concentrations under these conditions.

### 6. Determination of the mitochondrial affinity for ADP

All experimental data were treated with a specific homemade program developed using the Igor Pro software (Wavemetrics). As both oxidation and phosphorylation rates were simultaneously determined for each ADP concentration, mitochondrial apparent Km for ADP could be determined for both oxidation (K_mVox_) and phosphorylation (K_mVp_) rates. K_mVox_ and K_mVp_ for ADP were determined by fitting respectively oxidation and phosphorylation rates expressed as a function of the ADP concentration by the Michaelis-Menten equation (eq. 3):
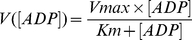
(3)where [ADP] corresponds to a measured ADP concentration, V to the corresponding oxidation or phosphorylation rate, Vmax to the maximal oxidation or phosphorylation rate and apparent K_m_ corresponds to the Michaelis-Menten constant.

### 7. Statistics

Experimental values are expressed as mean ± SD. Comparison between liver and muscle mitochondria were performed using unpaired bilateral student's t-tests. p-values were fixed at 0.05 and 0.01 to consider a significant level of difference between series of data.

## Results and Discussion

Mitochondrial oxidative phosphorylation involves complex biochemical processes in close interactions. The complete and accurate study of mitochondrial bioenergetics is therefore a technical challenge, especially in order to obtain physiologically relevant information (e.g. under low ADP concentrations). To face this challenge, we combined and adapted existing methods in an original way to investigate mitochondrial bioenergetics and its regulation by ADP. To demonstrate the discriminating capabilities of the proposed method, we chose to compare mitochondria isolated from skeletal muscle and liver (classically used as model mitochondria). Indeed, it has been shown that mitochondrial oxidative phosphorylation significantly differs in these two tissues [24] and these differences have been proposed to result from the distinct physiological roles of these organs [25].

### 1. Simultaneous determination of oxidation and phosphorylation rates during true steady states

The complete study of mitochondrial oxidative phosphorylation cannot be restricted to respiratory chain activity. Indeed, due to proton leak dependency on membrane potential (Δψ), the degree of coupling between oxidation and phosphorylation is known to largely vary depending on the rate of ATP synthesis and Δψ [16], [17], [26]. This coupling is raised up as the ATP synthesis rate increases since Δψ decreases. Respiratory chain activity is therefore not directly representative of phosphorylation activity, especially under low ADP concentrations, where the rate of ATP synthesis is low and Δψ is high. As a consequence, a better understanding of oxidative phosphorylation could be obtained by the concomitant determination of both oxidation and phosphorylation rates. In addition, the investigation of mitochondrial regulation by ADP in isolated mitochondria requires (i) a precise determination of oxidation and phosphorylation rates, especially at low ADP concentrations where oxidation and phosphorylation rates are low, and (ii) the control and accurate measurement of ADP concentration accessible to mitochondria during experiments. These technical difficulties can only be overcame by studying mitochondrial oxidative phosphorylation during true steady states, e.g. constant ATP turnover, where oxidation rate, phosphorylation rate and nucleotides concentrations are stable.

The glucose-hexokinase enzymatic system is widely used as ADP-regenerating system [27], [28] and was therefore chosen to establish true steady states of oxidative phosphorylation. In addition, this enzymatic system was coupled to G6PDH-NADP^+^ in order to determine the phosphorylation rate, since NADPH produced is stoichiometrically linked to mitochondrial ATP production [23] (see materials and methods for details). By placing in the oxygraphic vessel an optic fiber connected to a spectrometer in order to monitor changes in NADPH concentration, oxidation and phosphorylation rates were therefore determined simultaneously. Since a constant ATP turnover may be established by this coupled enzymatic system, various phosphorylation activities can be easily set-up with decreased experimental errors, and the study of oxidative phosphorylation under low ADP concentrations becomes accessible and accurate. By taking samples during each recording, both ADP and ATP concentrations were determined using a luciferine-luciferase based assays (see materials and methods for details).

**Figure 2:**
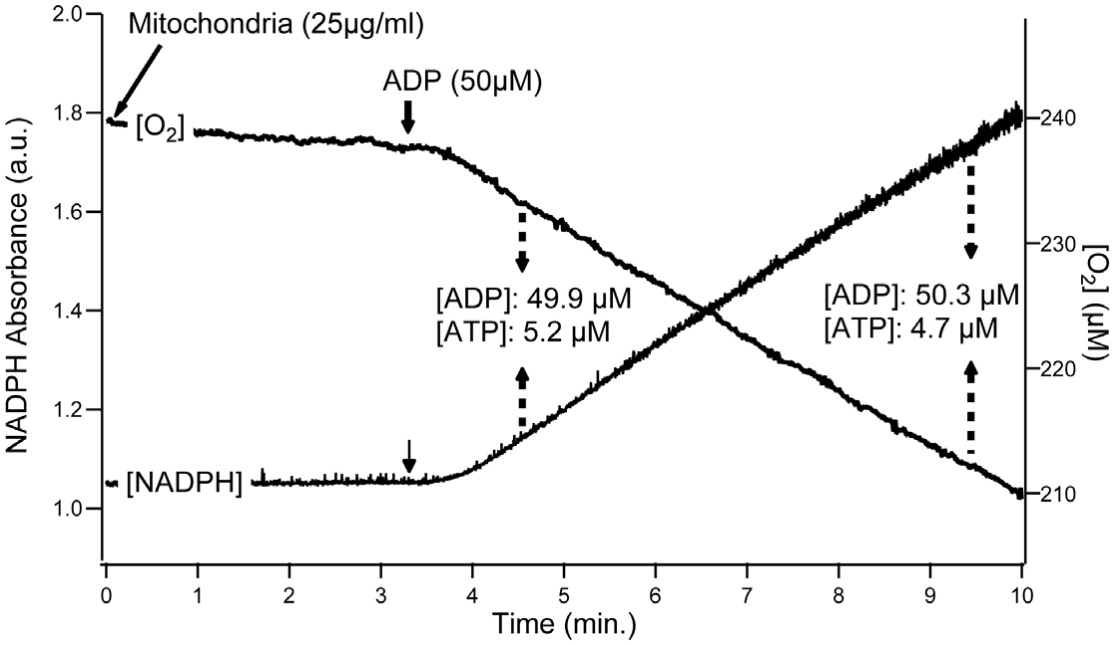
Typical recording of oxidation rate, phosphorylation rate and ADP/ATP concentrations. Oxidation and phosphorylation rates were recorded simultaneously in liver and muscle mitochondria oxidizing glutamate+malate+succinate as substrates. Mitochondrial protein concentration in the oxygraphic vessel was 25 µg.ml^−1^. Steady states of oxygen consumption and phosphorylation rates were obtained using the coupled enzymatic system composed of Glucose (5 mM) - Hexokinase (2.5 U.ml^−1^, Sigma-Aldrich, H4502) - Glucose-6-phosphotate dehydrogenase (2.5 U.ml^−1^, Sigma-Aldrich, G6378) - NADP^+^ (1.6 mM). Dashed arrows correspond to the sampling of measurement medium taken from the oxygraphic vessel during each recording for determination of ADP and ATP concentrations.

Figure 2 shows a combined typical recording obtained using Glutamate+Malate+Succinate substrates in order to reconstitute the tricarboxylic acid cycle function and consequently to approach physiological conditions [29]. Mitochondria were added at the onset of the recording and quickly reached state 4 oxidation rate. In this example, the addition of 50 µM ADP triggered phosphorylation, leading to a corresponding increase in oxidation rate. Figure 2 shows that both ADP-induced oxidation and phosphorylation rates were constant during the duration of the recording (approximately 6 min). In order to determine ADP and ATP concentrations, two samplings were performed at the beginning and at the end of recording. As it can be seen in figure 2, the stability of these two concentrations demonstrates the achievement of true steady states during the time course of the experiment.

### 2. Measurement of mitochondrial oxidation and phosphorylation affinity for ADP

Mitochondrial affinity for ADP can be investigated by determining the apparent K_m_ for ADP, a parameter that is related to the mitochondrial capacity to respond to changes in ATP demand and primarily reflects the kinetic properties of both the adenine nucleotide translocator and the F0-F1 ATP-synthase.

**Figure 3:**
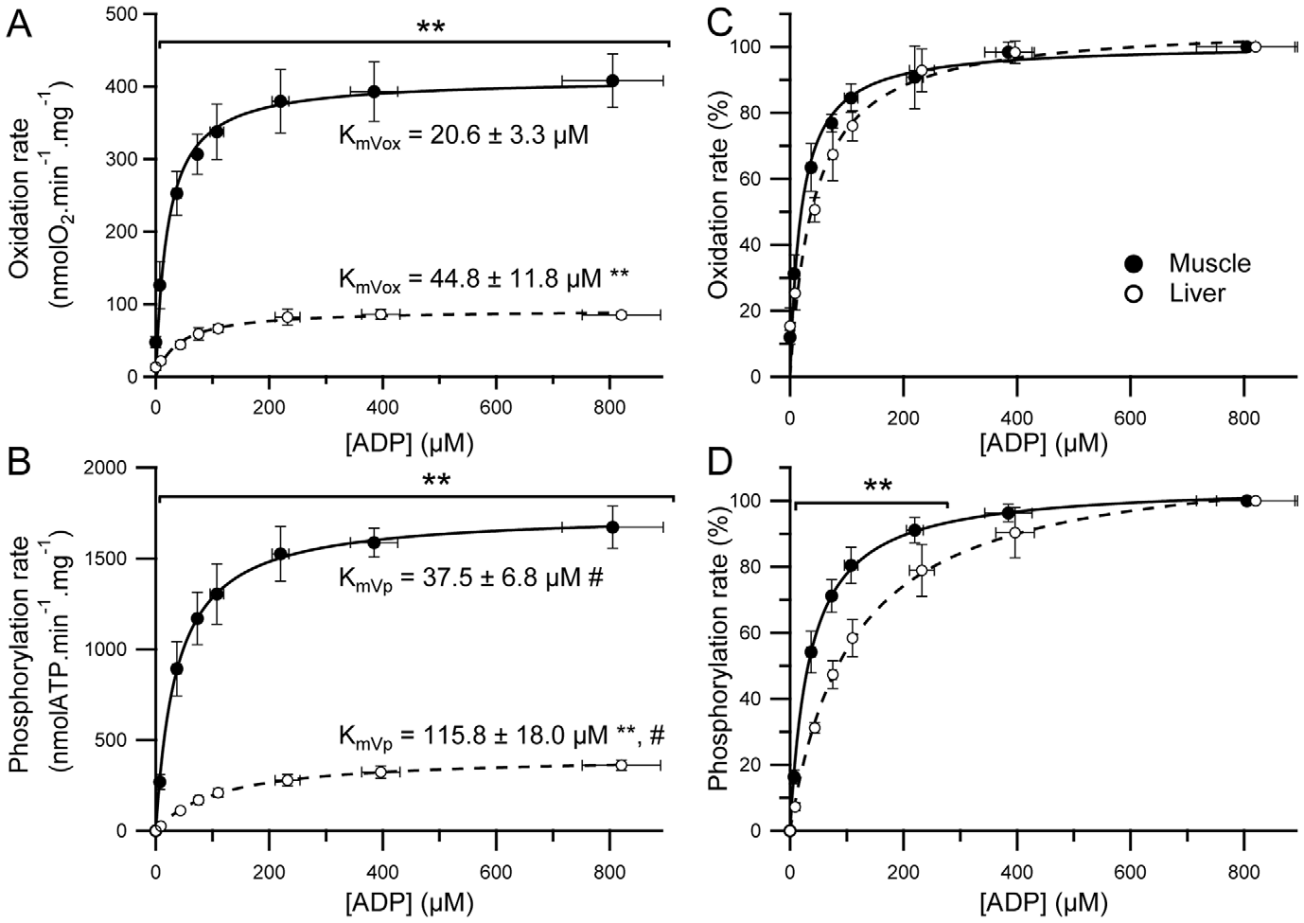
Dependence of oxidation and phosphorylation rates on ADP concentration in liver and muscle mitochondria. Oxidation and phosphorylation rates were recorded simultaneously in liver (n = 4) and muscle (n = 5) mitochondria oxidizing glutamate+malate+succinate as substrates. True steady state of oxidation and phosphorylation rates were obtained using coupled enzymatic system composed of Glucose (5 mM) - Hexokinase (2.5 U.ml^−1^, Sigma-Aldrich, H4502) - Glucose-6-phosphotate dehydrogenase (2.5 U.ml^−1^, Sigma-Aldrich, G6378) - NADP^+^ (1.6 mM). Data presented in panels A and B correspond to absolute oxidation and phosphorylation rates, respectively. Maximal oxidation and phosphorylation rates obtained for muscle and liver mitochondria were 408.0±42.5 vs. 85.2±5.5 nmolO_2_.min^−1^.mg^−1^ and 1672.8±134.1 vs. 361.3±33.41 nmolATP.min^−1^.mg^−1^, respectively. Panels C and D show normalized oxidation and phosphorylation rates, respectively. Data were fitted using the Michaelis-Menten equation presented in the materials and methods section. Data are presented as mean ± SD. Differences were tested using an unpaired bilateral student's t-test. ** p<0.01 between liver and muscle, # p<0.01 vs. KmVox.

In the present study, both oxidation and phosphorylation rates were therefore determined as described above during steady states under measured ADP concentrations ranging from 7 to 900 µM. The dependence of oxidation and phosphorylation rates on ADP concentration for liver and muscle mitochondria are shown in figure 3. Maximal oxidation and phosphorylation rates were more than four times higher in muscle as compared to liver mitochondria (figure 3 A and B). These results are in accordance with previously shown higher state III oxidation rate in muscle mitochondria [24]. In addition, it is interesting to note that even for the very low ADP concentrations used in the present study, both oxidation and phosphorylation rates were significantly much higher in muscle mitochondria (figure 3A and B). To our knowledge, these oxidation and phosphorylation rates kinetics allowed for the first time the accurate determination of K_mVox_ and K_mVp_. As shown in figure 3A and B, mitochondria isolated from skeletal muscle were characterized by significantly lower K_mVox_ and K_mVp_ as compared to liver mitochondria, clearly indicating a much higher affinity for ADP in muscle mitochondria.

Interestingly, the K_mVp_ was significantly higher as compared to K_mVox_ in both tissues (figure 3 A and B). During *in vitro* experiments, the onset of phosphorylation caused by ADP induces a decrease in Δψ, which in turn decreases proton leak and increases respiratory chain activity. The more the phosphorylation rate is high, the more Δψ decreases and consequently the more proton leak decreases [16], [17], [26]. The contribution of the oxygen consumption due to non-phosphorylating processes (e. g. proton leak) in the global activity of the respiratory chain therefore decreases as phosphorylation rate increases. Consequently, the determination of K_mVox_ leads to a clear overestimation of mitochondrial affinity for ADP. The accurate determination of mitochondrial oxidative phosphorylation affinity for ADP therefore requires the determination of the apparent K_m_ for ADP from phosphorylation rates (K_mVp_). This can easily and accurately be done in isolated mitochondria using the proposed method.

In addition, this method allows the comparison of the kinetics of oxidation and phosphorylation rates in response to changes in the ADP concentration between liver and muscle mitochondria (figure 3 C and D). As shown in figure 3 C, liver and muscle mitochondria presented only slight differences when compared to those obtained when considering phosphorylation rate kinetics (figure 3 D). It can be seen in figure 3 D that phosphorylation rate is always more responsive in muscle mitochondria as compared to liver for a given ADP concentration. In accordance with these results, the K_mVp_ to K_mVox_ ratio was significantly higher in liver mitochondria (2.7±0.4 vs. 1.8±0.4, p<0.05). Taken all together, these results emphasize the interest of the determination of both K_mVox_ and K_mVp_, since the information provided by each of these parameters are complementary in order to characterize the integrated functioning of oxidative phosphorylation. In addition, major functional differences in the phosphorylation rate response to an increase in the ADP concentration were revealed under low ADP concentrations (figure 3 D), which are concentrations likely to occur *in vivo*. Interestingly, the proposed method ensures a high degree of precision in the determination of both oxidation and phosphorylation rates for these ADP concentrations.

### 3. Determination of mitochondrial coupling efficiency

**Figure 4:**
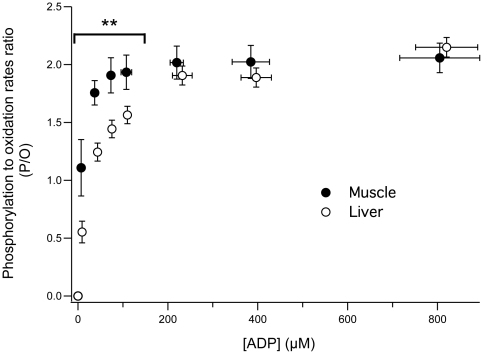
Changes in the P/O ratio as a function of ADP concentration in liver and muscle mitochondria. P/O ratio was determined by calculating the phosphorylation to oxidation rates ratio. Data for liver (n = 4) and muscle (n = 5) are presented as mean ± SD. Differences were tested using an unpaired bilateral student's t-test. ** p<0.01 between liver and muscle.

The coupling efficiency of oxidative phosphorylation can be defined as the amount of ATP molecules that mitochondria can synthesize for each atom of oxygen consumed. This coupling efficiency is central to the physiology of energy metabolism and can be assessed by the determination of the well-known P/O ratio [27], [30]. Since both oxidation and phosphorylation rates are determined simultaneously using the proposed method, this crucial parameter is directly accessible. P/O ratio was therefore calculated for every ADP concentration (figure 4). Maximal P/O ratio, obtained under the highest ADP concentrations (from 200 to 900 µM), was similar between liver and muscle mitochondria. However and most interestingly, under low ADP concentrations (from approximately 7 to 150 µM), the P/O ratio was significantly higher in muscle mitochondria. According to this result, under low ADP concentrations, muscle mitochondria synthesize a higher amount of ATP for the same amount of oxygen as compared to liver mitochondria. In this way muscle mitochondria appear optimized toward ATP production at a high yield even at low ADP concentrations when compared to liver mitochondria.

These results demonstrate the effectiveness of the proposed method in the determination of changes in P/O value in response to an increase in ADP concentration. Moreover, as compared to other available methods, the direct determination of both oxidation and phosphorylation rates ensures a more accurate calculation of P/O value.

### Conclusions

The present paper demonstrates that the correct determination of mitochondrial oxidative phosphorylation affinity for ADP in isolated mitochondria is accessible using the proposed method.

The application of the method described in the present paper enabled to emphasize major differences between muscle and liver mitochondrial bioenergetics. Compared to liver, muscle mitochondria were characterized by a higher oxidative phosphorylation affinity for ADP, associated with a higher P/O ratio under low ADP concentrations and a higher maximal capacity to produce ATP. Consequently, muscle mitochondria appear more designed to better respond to any variation in the ATP demand in contrast to liver mitochondria certainly more designed to assume their metabolic crossroads function.

When using the method presented here, phosphorylation rates are directly determined during true steady states, making consequently the determination of oxidative phosphorylation affinity for ADP accurate. The concomitant determination of the corresponding oxidation rates also brings crucial information since P/O ratio can be calculated. As a consequence, any modification of the inner mitochondrial membrane properties (i.e. modification of proton leak) may be revealed after application of the present method. In addition, the direct determination of both ADP and ATP concentrations increases the precision in the determination of mitochondrial affinity for ADP. This method consequently represents an easy way to obtain a complete and accurate check-up of integrated mitochondrial bioenergetics with a special emphasis on low ADP concentrations, likely to occur under *in vivo* conditions. In addition, since the amount of mitochondrial proteins required to determine all these bioenergetics parameters is relatively low (approximately 200–250 µg), this method could therefore be applied to study mitochondrial bioenergetics in small amount of tissue from knock-out mice models or in human biopsies.

Our method could therefore be of some importance to determine whether mutations or alterations demonstrated at the molecular level can lead or not to true functional alterations. This method therefore opens the route to a better understanding of functional consequences of mitochondrial dysfunctions arising in pathologies.
